# Investigating the Therapeutic Mechanisms of Total Saikosaponins in Alzheimer’s Disease: A Metabolomic and Proteomic Approach

**DOI:** 10.3390/ph18010100

**Published:** 2025-01-15

**Authors:** Huiling Wei, Tianyi Du, Weiwei Zhang, Wei Ma, Yao Yao, Juan Li

**Affiliations:** 1 School of Basic Medical Sciences, Ningxia Medical University, Yinchuan 750004, China; 19151918756@163.com (H.W.); m18937343116@163.com (T.D.); 20190033@nxmu.edu.cn (W.Z.); mwei163@126.com (W.M.); 2 School of Pharmacy, Ningxia Medical University, Yinchuan 750004, China; 3 Ningxia Engineering and Technology Research Center for Modernization of Characteristic Chinese Medicine, and Key Laboratory of Ningxia Ethnomedicine Modernization, Ministry of Education, Ningxia Medical University, Yinchuan 750004, China

**Keywords:** Alzheimer’s disease, saikosaponins, metabolomics, proteomics, biomarkers

## Abstract

Alzheimer’s disease (AD) is the leading cause of dementia among the elderly, yet effective treatments remain elusive. Total saikosaponins (TSS), the primary bioactive components in *Bupleurum chinense*, have shown promising therapeutic effects against AD in previous studies. **Methods**: To delve deeper into the mechanisms underlying the therapeutic role of TSS in AD, we investigated its neuroprotective effects and associated molecular mechanisms in APP/PS1 mice. Further, we employed metabolomic and proteomic analyses, with a focus on the potential protein-level changes induced by TSS, particularly those related to metabolite accumulation in the brain. **Results**: Our results showed that lysophosphatidylcholine, adenosine, and sphingomyelin in plasma might serve as potential biomarkers. Compared to the control group, AD mice exhibited significantly increased expression of proteins related to neuroinflammatory pathways, whereas proteins involved in cAMP signaling, cGMP-PKG signaling, and synaptic plasticity pathways were significantly downregulated. Notably, these signaling pathways were partially reversed in APP/PS1 mice following TSS administration. Behavioral tests demonstrated that TSS effectively improved the learning and memory functions of mice. **Conclusions**: Our findings suggest that TSS ameliorate cognitive decline through regulating neuroinflammatory pathways, cAMP and cGMP signaling, and synaptic plasticity pathways, providing insights into its therapeutic potential in AD.

## 1. Introduction

Alzheimer’s disease (AD) is a common cause of cognitive impairment and dementia in the elderly population. Its core pathological hallmarks include the extracellular accumulation of β-amyloid (Aβ) and intraneuronal neurofibrillary tangles enriched with tau protein [1,2]. These Aβ and tau proteins initiate a cascade of reactions leading to neural circuit damage and cognitive decline [3,4]. Currently, there are approximately 50 million cases of AD worldwide. With the increasing elderly population, it is projected that the global prevalence of neurodegenerative dementia will rise to 113 million by 2050 [5,6]. As early as 1992, Aβ was confirmed to exist in cerebrospinal fluid (CSF), and biomarker development for AD has mainly focused on CSF, with the ratio of Aβ40/Aβ42 in CSF being assessed using positron emission tomography (PET) scans [7,8,9]. However, these methods are invasive and painful [10], making blood an ideal sample source for screening tests. Through the use of plasma and serum, several metabolites associated with inflammation, oxidative stress, mitochondrial dysfunction, and neuronal damage have been identified in AD [11,12]. Given the complexity of AD pathogenesis, there is currently no cure for this disease. Therefore, research and development aimed at discovering novel therapeutic strategies are crucial.

*Radix bupleuri* (Chaihu), a commonly used traditional Chinese medicine, has been shown to possess neuroprotective, anti-inflammatory, anti-cancer, and immunomodulatory effects [13]. Traditional formulas such as Chaihu Shugan San and Xiao Chaihu Tang, which contain *Radix bupleuri* as the main ingredient, have been used in the treatment of AD and found to improve cognitive impairment in AD patients [14,15,16]. Saikosaponins, pentacyclic triterpenoid oleanane-type derivatives, are the primary active components of *Radix bupleuri*. Recently, dozens of saikosaponins have been isolated from *Radix bupleuri*, with saikosaponin a and d being the most abundant [17]. Our previous studies have demonstrated that total saikosaponins (TSS) can ameliorate cognitive dysfunction, reduce Aβ production and senile plaque deposition, and inhibit neuroinflammation in APP/PS1 transgenic mice by downregulating the expression of β-secretase 1 (BACE1) and activating Nrf2 [18]. Furthermore, TSS have the capacity to mitigate synaptic loss and oxidative stress. Studies have further demonstrated that saikosaponin c effectively inhibits the secretion of both Aβ_1-40_ and Aβ_1-42_ peptides, as well as the abnormal phosphorylation of tau proteins at multiple residues implicated in AD [19,20]. This action facilitates neurotrophin-induced neurite outgrowth. In conclusion, saikosaponins exhibit promising therapeutic effects on AD, but the underlying mechanisms require further elucidation.

**Figure 1:**
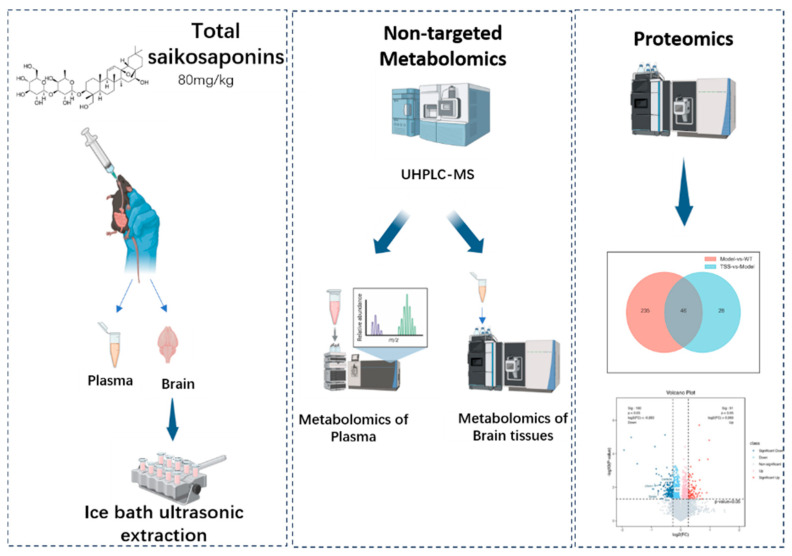
Workflow diagram.

In this study, the therapeutic effect and related molecular mechanism of TSS in APP/PS1 mice were investigated. Behavioral experiments showed that TSS could effectively improve the learning and memory function of AD mice. To further investigate the underlying mechanisms of the anti-AD effects of TSS, we conducted comprehensive metabolomic and proteomic studies utilizing liquid chromatography–mass spectrometry (LC-MS) workflows. These workflows leveraged the high sensitivity, resolution, and extensive coverage of metabolites and peptides, ensuring a thorough analysis [21]. Metabolomic and proteomic analyses were performed on plasma and brain tissues derived from wild-type mice, APP/PS1 transgenic mice, and TSS-treated transgenic mice. These analyses were carried out using a streamlined preparation process and high-resolution mass spectrometry techniques, such as ultra-high-performance liquid chromatography–mass spectrometry (UHPLC-MS) and liquid chromatography–tandem mass spectrometry (LC-MS/MS) to ensure precise and detailed insights. The workflow of this study is shown in Figure 1.

## 2. Results

### 2.1. TSS Treatment Can Alleviate Cognitive Dysfunction in APP/PS1 Mice

**Figure 2:**
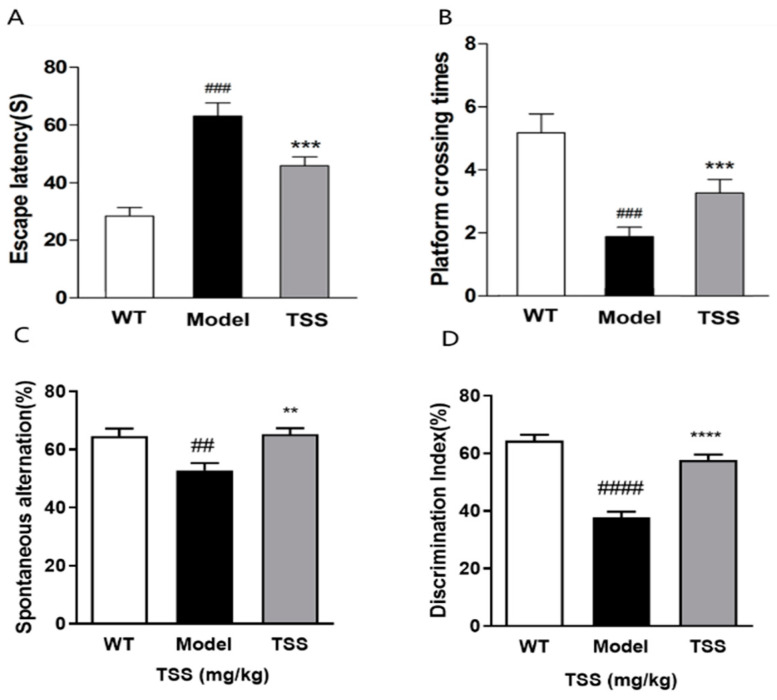
TSS ameliorate cognitive dysfunction in AD mice: (**A**) the Morris water maze was used to investigate the effect of TSS on escape latency in AD mice (### *p* < 0.001, *** *p* < 0.001); (**B**) the effect of TSS on the number of platform crossing times in AD mice was investigated in the Morris water maze test (### *p* < 0.001, *** *p* < 0.001); (**C**) the effect of TSS on the residence time of AD mice was assessed with the Y-maze test (## *p* < 0.01, ** *p* < 0.01 ); (**D**) the NOR test was used to investigate the effect of TSS on the recognition index in AD mice (#### *p* < 0.0001, **** *p* < 0.0001). # Comparison with WT group; * comparison with model group. Data are expressed as mean ± S.E.M. (n = 6).

The impact of TSS on spatial memory impairment in APP/PS1 transgenic mice was evaluated through the Morris water maze (MWM) test (Figure 2A,C). Compared to wild-type (WT) mice, APP/PS1 mice exhibited a significant increase in escape latency (Figure 2A, *p* < 0.001) during the place navigation test and a notable decrease in the platform crossing numbers during the spatial probe test (Figure 2B, *p* < 0.001), indicating severe spatial memory impairment. Compared to the model group, the TSS-treated group (80 mg/kg) demonstrated a significant reduction in escape latency (Figure 2A, *p* < 0.05) and a notable increase in platform crossings (Figure 2B, *p* < 0.05), suggesting that TSS treatment significantly ameliorated spatial memory impairment in APP/PS1 mice.

A Y-maze test was employed to assess the effect of TSS on spatial working memory impairment in AD mice. Compared to the WT mice, the model group exhibited a significant decrease in the spontaneous alternation rate (Figure 2D, *p* < 0.01), indicating impaired spatial memory. Notably, following TSS treatment, the spontaneous alternation rate of AD mice increased significantly (Figure 2D, *p* < 0.01), suggesting that TSS are effective in enhancing spatial working memory in AD mice.

The impact of TSS on novel object recognition (NOR) deficits in APP/PS1 mice was evaluated through the NOR test. The results revealed that the discrimination index (DI) of the model group was markedly lower than that of WT mice (Figure 2C, *p* < 0.0001). After TSS treatment, the DI significantly increased, suggesting that TSS improved the NOR ability of the model group (Figure 2D, *p* < 0.0001). In summary, these findings indicate that TSS treatment substantially alleviated cognitive dysfunction in the model group.

### 2.2. Metabolomic Analysis

#### 2.2.1. Reliability Assessment of the Analytical Method

**Figure 3:**
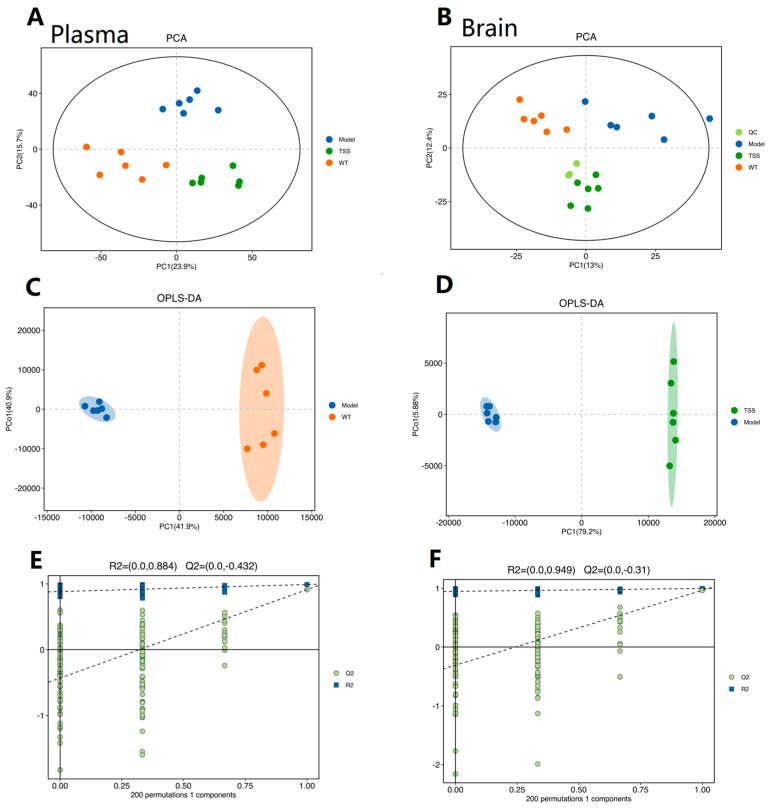
Metabolic score of plasma (**A**) and brain tissue (**B**). The PCA score diagram of 6 groups of QC samples showed that the sample distribution was concentrated, indicating that the sample had high stability and good repeatability. Model vs. WT (**C**,**E**); TSS vs. Model (**D**,**F**).

In both plasma and brain tissue samples, all six quality control (QC) samples were consistently positioned in the center of the principal component analysis (PCA) score plot. This result suggests that the established method exhibits excellent reproducibility and system stability (Figure 3A,B). During the analysis, abnormal samples were removed, resulting in a well-fitted OPLS-DA model with VIP > 1, demonstrating the strong estimation and prediction capabilities of the model for the first two principal components.

Furthermore, to ensure that the orthogonal partial least square–discriminant analysis (OPLS-DA) model did not yield classifications by chance through supervised learning methods, it underwent 200 permutation tests, confirming that Q2 < 0. Importantly, all green Q2 values on the left side of the plot were lower than the original points on the right side, indicating that the constructed model has high goodness of fit and robust predictive ability (Figure 3C–F).

#### 2.2.2. Differential Metabolite Screening and Enrichment Analysis

**Figure 4:**
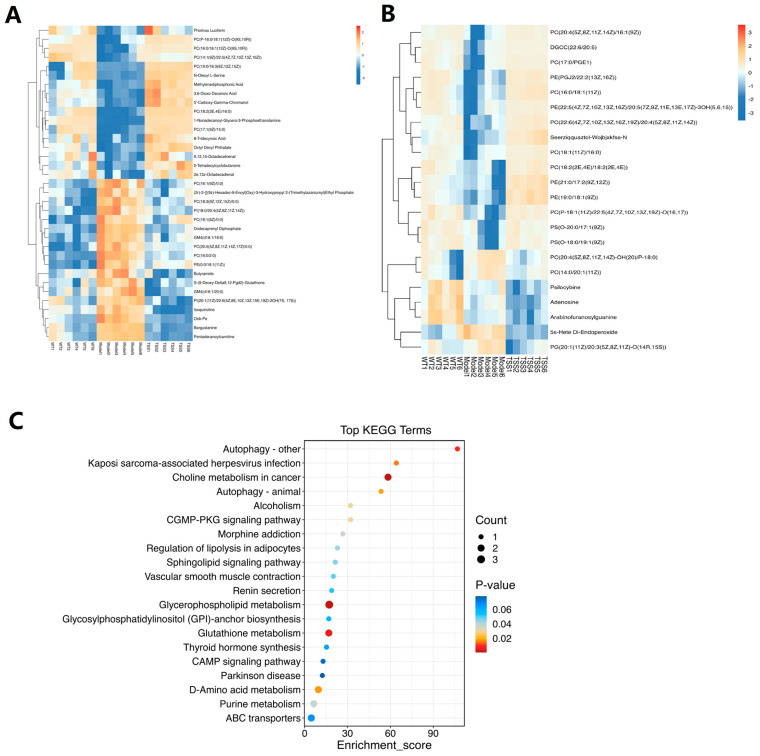
Plasma metabolites cluster heat map (**A**) and brain tissue metabolites cluster heat map (**B**); the blue color indicates reduced metabolites and red indicates elevated metabolites. KEGG enrichment analysis of metabolites (**C**); the color of the circle represents the number of metabolites enriched. A darker color indicates a greater degree of enrichment. The size of the circle represents the number of metabolites enriched in the metabolic pathway. A larger circle indicates a greater number of metabolites.

Fifty-four differential metabolites were identified, including adenosine, phosphatidylcholine, choline, ethanolamine, fatty amino acids, oleamides, sphingolipids, glutathione, phospholipids, and other metabolites. Among the metabolites identified in both plasma and brain tissue, we identified 35 overlapping key metabolites that were shared among the WT, model, and TSS groups in plasma (Figure 4A) and 22 overlapping key metabolites in brain tissue (Figure 4B). Following the integration of all the identified differential metabolites (Table 1 and Table 2), hierarchical clustering analysis was performed based on the levels of the top 50 most significantly different metabolites, which had high variable importance in projection (VIP) scores. Analysis of the heat maps revealed the upregulation and downregulation of metabolites in plasma when compared pairwise. Specifically, when compared to the model group, the plasma of the TSS group exhibited 31 distinct metabolites, with 16 being upregulated and 15 being downregulated (Figure 4A). Meanwhile, in brain tissue, the TSS group showed 15 upregulated metabolites and 4 downregulated metabolites compared to the model group (Figure 4B).

The small-molecule metabolites involved in sphingolipid metabolism in plasma are primarily regulated by TSS, with *p*-values < 0.05 and FC > 1.2. Among them, five phosphatidylcholines are decreased in AD plasma, while significant changes in phospholipid and sphingolipid metabolism also occur in the blood of AD patients [22]. Sphingolipids play a crucial role in the pathogenesis of inflammation induced by amyloid-β and proinflammatory cytokines in AD [23]. Furthermore, metabolomic analysis revealed an elevation of choline in AD. It is well established that the cholinergic system plays a crucial role in learning and memory processes, and it has been reported that serum choline levels undergo a notable decrease following treatment. Our findings underscore the significant potential of phospholipids to serve as a biomarker for AD.

To explore the pathways influenced by TSS, we annotated the TOP20 pathway metabolites according to KEGG (Figure 4C). Our study revealed that the majority of the metabolites were implicated in alpha-linolenic acid metabolism, glycerophospholipid metabolism, linoleic acid metabolism, purine metabolism, arachidonic acid metabolism, cAMP signaling pathway, and cGMP-PKA signaling pathway. The results suggest that these metabolites and their associated metabolic pathways play pivotal roles in the therapeutic effect of TSS in AD mice.

### 2.3. Proteomic Analysis

**Figure 5:**
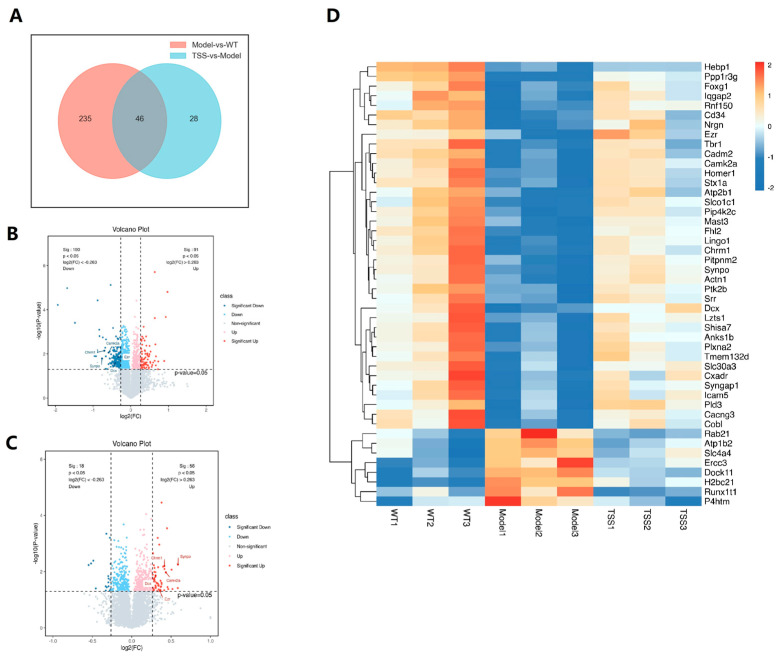
Proteomic analysis of brain tissue: The Venn diagram (**A**); the volcano diagram is limited to *p*-value > 0.05 and FC < 1.2; each point represents one kind of protein, with red indicating upregulation and blue indicating downregulation (**B**,**C**); the heat map clustering of all detected proteins in the brain tissue of WT, model and TSS groups (**D**).

Based on the analysis of all 309 identified proteins, as shown in Figure 5A, 46 key proteins were found to overlap between the WT, model, and TSS groups. Clustering analysis provided an overview of the relative increases (brown) or decreases (blue) in protein levels across the WT, model, and TSS groups (Figure 5B). Subsequently, 46 significantly dysregulated proteins were selected (Table 3), including 9 downregulated and 37 upregulated proteins. Combining positive and negative data, 434 proteins with significant differences between the two groups were filtered out. The volcano plot graphically depicts the comparison of ion features between -log10 (*p*-value) and log2 (FC) (Figure 5C,D), with *p* > 0.05. Proteins that are upregulated are displayed on the right, while downregulated proteins are on the left. Red and blue colors in the plot represent upregulated and downregulated proteins, respectively, with darker colors indicating more significant differences. Gray points represent proteins with *p*-values ≥ 0.05. It is challenging to determine which protein is the most crucial target.

Furthermore, we found that TSS significantly regulated these differentially expressed proteins (DEPs), with 37 proteins upregulated and 9 proteins downregulated, comparing to the model group (*p* < 0.05). Distinct clustering patterns were observed in both treatment groups (Figure 5C,D). Six related potential protein biomarkers, namely PLD3, Camk2a, Synpo, Dcx, Ezr, and Chrm1, were all significantly decreased in the model group (*p* < 0.05). Additionally, through pairwise comparisons to identify the target proteins of the two treatments, we discovered that TSS significantly upregulated Chrm1 and Synpo. Further validation of protein and RNA level changes will require additional experiments such as Western blotting, qPCR, and immunofluorescence.

### 2.4. GO Pathway Analysis of DEPs

To gain a deeper understanding of the protein expression patterns through which TSS exert neuroprotective effects, we conducted an enhanced Gene Ontology (GO) functional analysis of the proteomic data. GO enrichment analysis was performed to elucidate the specific biological functions of the proteins through three comparisons: model vs. WT, TSS vs. model, and TSS vs. WT groups. These functions were systematically categorized into three distinct groups: biological processes (BPs), cellular components (CCs), and molecular functions (MFs).

**Figure 6:**
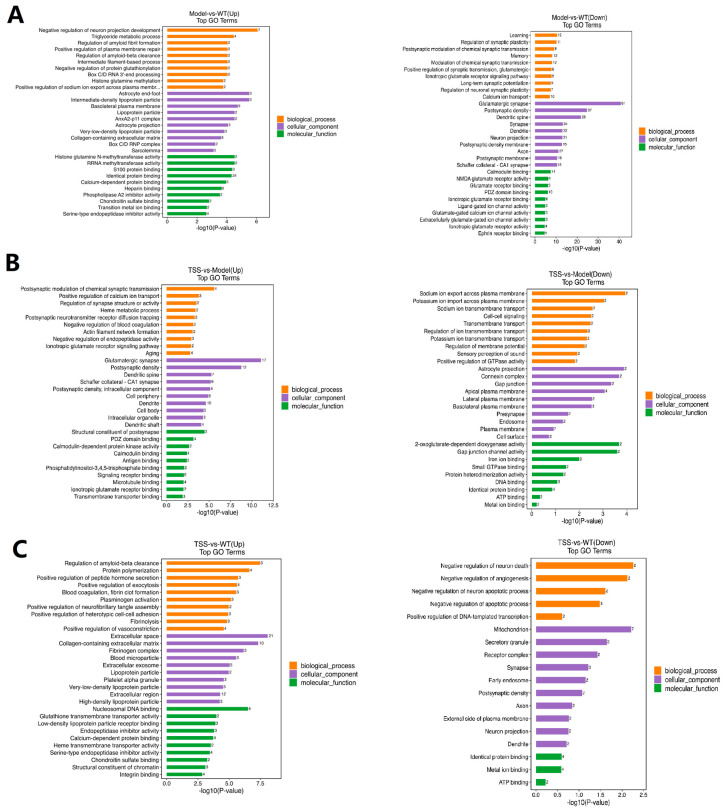
Gene Ontology (GO) enrichment analysis. The upregulated pathways are depicted on the left, and the downregulated pathways are depicted on the right: (**A**) the most enriched protein entries in the model and WT groups; (**B**) the most enriched protein entries in the TSS group and the model groups; (**C**) the most enriched protein entries in the TSS and WT groups. Orange represents biological processes (BPs), purple represents cellular components (CCs), and green represents molecular functions (MFs).

In the comparison between the model and WT groups, the upregulated proteins enriched according to the GO analysis were primarily involved in the positive regulation of biological processes (BPs) such as the negative regulation of neuron projection development, glycerolipid metabolic process, and the regulation of amyloid clearance. They were also enriched in cellular components (CCs) such as myofibrillar membrane and basolateral plasma membrane (Figure 6A). Downregulated proteins, on the other hand, participated in BPs like learning, memory, and the regulation of neuronal synaptic signaling. They were enriched in CCs related to calmodulin binding, glutamate receptor binding, and the regulation of NMDA glutamate receptor activity.

In the comparison between TSS and model groups, in terms of CCs, upregulated proteins were mainly enriched in extracellular regions and intracellular organelles, while regarding molecular functions (MFs), they were enriched in signal receptor binding and microtubule binding. In BPs, these proteins were involved in the postsynaptic modulation of chemical synapses and the positive regulation of calcium ion transport. Conversely, downregulated proteins were enriched in CCs such as the mitochondrion, the external side of the plasma membrane, the synapse, and the axon. In BPs, they were involved in the negative regulation of the neuronal apoptotic process and apoptotic process (Figure 6B).

In the comparison between the TSS group and the WT group, the results demonstrated that the regulation of amyloid clearance and protein polymerization were the most significantly enriched BP categories. Extracellular space and the extracellular matrix were primarily enriched in CC categories, while nucleosome DNA binding and calcium-dependent protein binding were enriched in MF categories (Figure 6C).

### 2.5. KEGG Enrichment Analysis of DEPs

**Figure 7:**
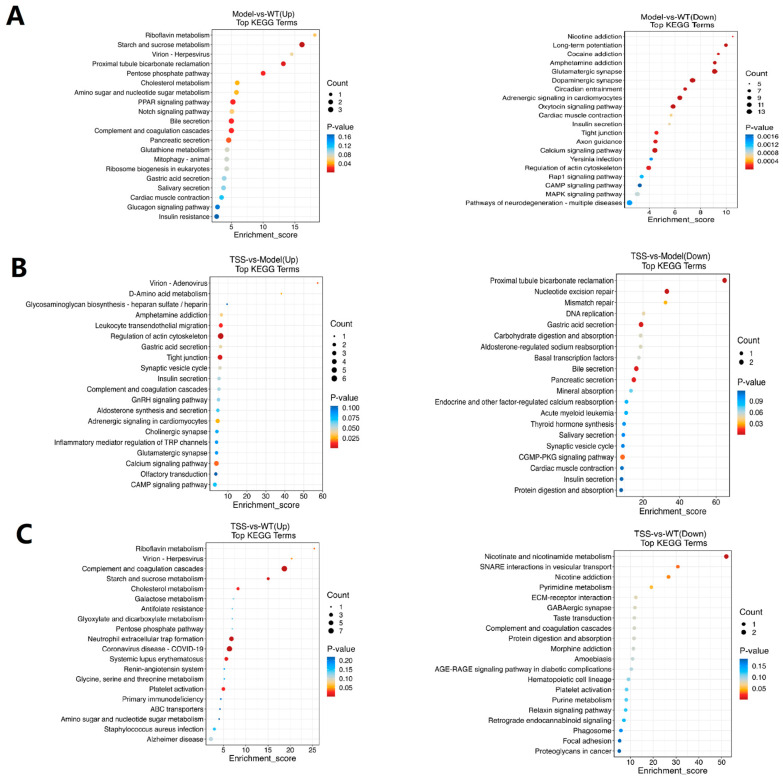
KEGG enrichment analysis of differentially expressed proteins (DEPs). The upregulated pathways are depicted on the left, and the downregulated pathways are depicted on the right: (**A**) pathway enrichment statistics of model vs. WT groups; (**B**) pathway enrichment statistics of TSS vs. model groups; (**C**) pathway enrichment statistics of TSS vs. WT groups. The color of the circle indicates the degree of enrichment. Darker colors signify a higher degree of enrichment. The size of the circle represents the number of proteins enriched in the metabolic pathway. Larger circles indicate a greater number of proteins.

These proteins were subjected to further classification utilizing KEGG pathway analysis. The results revealed that the majority of both upregulated and downregulated proteins were predominantly enriched in metabolic pathways, human diseases, genetic information processing, and cellular processes. Among them, pathways related to glutathione metabolism, PPAR signaling, bile secretion, and Notch signaling were the primary enriched pathways for upregulated proteins. In contrast, pathways related to cAMP signaling, cGMP-PKG signaling, glutamatergic synapse, and neurodegenerative diseases were the primary enriched pathways for downregulated proteins (Figure 7A).

In the comparison between the TSS and model groups, the upregulated KEGG pathways were associated with the regulation of the actin cytoskeleton, tight junction, and cAMP signaling. Meanwhile, the downregulated pathways were mainly associated with pancreatic secretion, nucleotide excision repair, bile secretion, and cGMP-PKG signaling (Figure 7B). Similarly, in the comparison between the TSS and WT groups, the upregulated proteins were primarily enriched in the regulation of the actin cytoskeleton, tight junction, calcium signaling, and cAMP signaling. On the other hand, the downregulated proteins were mainly enriched in nicotinate and nicotinamide metabolism (Figure 7C).

### 2.6. Comprehensive Analysis of Metabolomic and Proteomic Data

**Figure 8:**
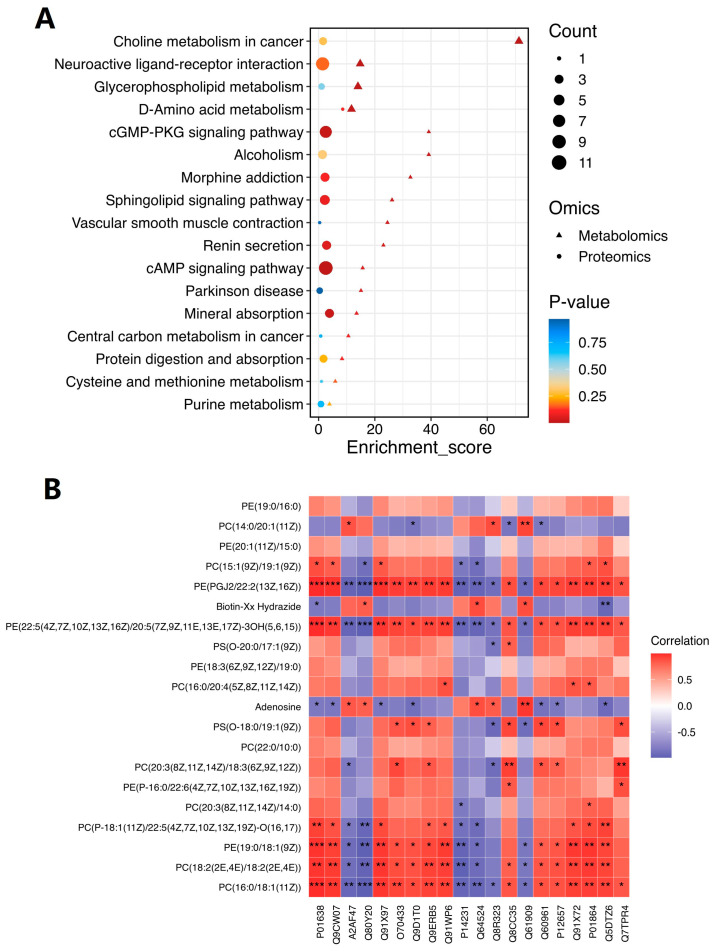
Combined analysis diagram of metabolomics and proteomics: (**A**) the KEGG bubble map was drawn by the common pathway of differential proteins and metabolites. The circles in the figure represent differential proteins, and the triangles represent differential metabolites. Larger circles/triangles indicate a greater number of differential proteins/metabolites contained in the pathway. The horizontal axis represents the enrichment score, while the vertical axis displays the common pathway information; (**B**) correlation analysis between differential proteins and differential metabolites. Red indicates a positive correlation, and blue indicates a negative correlation. Deeper color indicates greater correlation; *** represents *p* < 0.001, ** represents *p* < 0.01, and * represents *p* < 0.05.

In order to further clarify the relationship between metabolomic and proteomic results, we established an enrichment pathway that reflects the corresponding homogeneous expression of dem and deg. The most important pathways were cAMP-PKA and cGMP-PKG signaling pathways. As shown in Figure 8A, the majority of the proteins were involved in these two pathways, with a small percentage of metabolites also playing a role. The size of the circle represents the number of proteins. A larger circle indicates a higher number and vice versa. The triangle represents the number of metabolites. The larger the triangle, the more the number of metabolites, and vice versa. Figure 8B shows the correlation between proteins and metabolites. For each metabolite with different behaviors in the figure, each column corresponds to a protein. Red indicates a positive correlation, and blue indicates a negative correlation; the deeper the color, the greater the correlation. The correlations between Chrm1 and PC(18:2(2E,4E)/18:2(2E,4E)), PE(19:0/18:1(9Z)), PS(O-18:0/1:1(9Z)) and PE(PGJ2/22:2(13Z,16Z)) in brain tissue were significant, and the interaction between Chrm1 and these metabolites needs further verification.

## 3. Discussion

AD is the most prevalent form of dementia among the elderly population. It is characterized by neurodegeneration, which results in neuronal damage and death, cognitive decline, and memory loss [24]. The APP/PS1 transgenic mouse model is a commonly used AD mouse model that expresses mutations in the amyloid precursor protein (APP) and presenilin genes under the control of the neuron-specific Thy1 promoter. These genetic mutations expedite the formation of Aβ and tau pathology in familial AD, leading to an earlier onset of the disease. APP/PS1 mice begin to develop cerebral amyloid pathology as early as 6–8 weeks of age. By 9 months of age, Aβ accumulates in the cerebral cortex and hippocampus, accompanied by tau hyperphosphorylation and a reduction in synaptic density near Aβ plaques. This pattern remains stable over time [25,26]. Previous studies have employed 2-month-old male APP/PS1 mice for comparative metabolomic analysis to identify crucial metabolic mechanisms and protein biomarkers associated with AD. Additionally, 9-month-old female APP/PS1 mice have been used for proteomic analysis, which has shown that Aβ-derived diffuse plaque (ADCS)-derived extracellular vesicles can ameliorate neuronal damage, foster neurodevelopment, and safeguard cognitive function in AD mice [27,28]. The above results demonstrate that the APP/PS1 mouse model is suitable for conducting AD proteomic and metabolomic studies.

Saikosaponins have been reported to show potent anti-AD effects. Saikosaponin D can not only eliminate intrinsic free radicals but also induce the activity of endogenous antioxidant enzymes and the expression of HO-1. By activating PI3K and thereby promoting the nuclear translocation of Nrf2, it enhances the cellular antioxidant capacity, protecting SH-SY5Y cells from glutamate-induced oxidative cytotoxicity [29]. Saikosaponin B2 has been reported to enhance the nuclear translocation of HLH-30 in Aβ transgenic *C. elegans*, which in turn boosts autophagy and aids in the breakdown of Aβ [30]. Our previous study revealed that TSS displayed neuroprotective properties in APP/PS1 transgenic mice, protecting cognitive function and mitigating neuropathological damage [18]. When administered at a dose of 80 mg/kg, TSS could improve cognitive impairments, decrease the production of Aβ, and diminish the accumulation of senile plaques. Furthermore, they reduce *p*-tau levels, oxidative stress, and inflammatory responses while inhibiting glial cell activation and promoting synaptic function. To elucidate the underlying therapeutic mechanisms of TSS on AD, we employed metabolomic and proteomic analyses with a focus on the potential metabolite- and protein-level changes induced by TSS.

The cognition protective effect of TSS was elucidated by behavioral assessments. A comprehensive analysis of metabolomic and proteomic data was employed to examine the global regulation of protein expression and metabolite accumulation. The behavioral results demonstrated that TSS treatment significantly enhanced cognitive function in APP/PS1 mice. Metabolomic analysis revealed a disruption in the purine metabolic pathway within the brains of APP/PS1 mice. In recent years, there has been limited understanding of the role of purine metabolism in AD. Further research in this area is needed. Adenosine, which is the most critical intermediate metabolite in the purine metabolic pathway, has been studied for its neuroprotective effects in AD. Adenosine is recognized as an extracellular signaling molecule that plays a crucial role in regulating, integrating, and fine-tuning neuronal activity. It influences brain functions such as sleep and wakefulness, cognition, and memory, as well as neuronal injury and degeneration [31]. Lysophosphatidylcholine and sphingolipids in plasma have been identified as potential biomarkers for AD. Pratishtha et al. determined plasma phospholipids and sphingolipids in non-carriers (NCs) and PSEN1 mutant carriers (MCs) [32]. The results showed that the significantly changed species in the spectrum were mainly cholines and ethanolamines, containing phospholipids and ceramides. In addition, in the MC group, phosphatidylcholine species (34:6, 36:5, 40:6) were associated with CSF tau, while phosphatidylcholine species (34:2, 36:4) were associated with CSF tau and the brain amyloid load. In the metabolomic analysis of APP/PS1 mice, we found that five phosphatidylcholines were downregulated in AD plasma and two phosphatidylcholines were downregulated in brain tissue, which were upregulated after TSS treatment.

Previous reports have highlighted the essential function of the cAMP/PKA signaling cascade in maintaining neuronal viability and facilitating the establishment of long-lasting memories [33,34]. Both cAMP and cGMP function as vital signaling molecules that are indispensable for long-term potentiation (LTP) and the consolidation of memories. However, the toxic effects of Aβ can impair the cAMP-PKA signaling pathway in AD [35,36]. Furthermore, the cAMP/PKA signaling pathway may also exacerbate the pathological process of AD by promoting Aβ accumulation and neuronal apoptosis [37]. Alterations in cAMP signaling within particular areas of the brain may be associated with the development of dementia. A notable decrease in cAMP signaling is a key factor contributing to the pathology of AD. Intriguingly, enhanced cAMP signaling in specific brain regions has been shown to mitigate age-related declines in brain function. Furthermore, research has indicated that the levels of cAMP in the hippocampus decreased through the overexpression of β-site amyloid precursor protein cleaving enzyme 1 (BACE1) or the introduction of Aβ_1-42_. These findings suggest that the modulation of cAMP signaling may represent a promising therapeutic approach for the treatment of AD [31,38]. PKG and cGMP are pivotal players in neuroinflammatory processes. These processes can result in impaired neurofunctional performance, cellular demise, and progressive neurodegeneration. By regulating various inflammatory signaling cascades, PKG and cGMP significantly impact neuronal health and survival, making them critical targets for understanding and mitigating neurodegenerative diseases [35]. Elevated cGMP levels can reduce the Aβ load in APP/PS1 mice, and the cGMP-PKG signaling pathway can protect neurons from Aβ-induced damage [39,40]. The cGMP-PKG signaling pathway plays a vital role in preventing neuronal apoptosis and promoting neuronal survival. When the activity of PKG is inhibited in hippocampal neurons, it can partially counteract the survival-enhancing effects of soluble amyloid precursor proteins (APPs). This suggests that cGMP may exert its neuroprotective effects through the activation of PKG, thereby highlighting the importance of this signaling pathway in maintaining neuronal health and viability [41]. Studies have confirmed that the pathophysiology of AD can be modulated by influencing the cGMP-PKG signal transduction pathway [42]. In our study, metabolomic analysis revealed significant metabolic enrichment in the cAMP-PKA and cGMP-PKG signaling pathways. Additionally, proteomic analysis revealed that the cAMP-PKA and cGMP-PKG signaling pathways were notably suppressed in the model group but exhibited significant upregulation following the administration of TSS. These findings suggest that these two signaling pathways may play crucial roles in the therapeutic mechanism of TSS in AD mice.

Proteomic analysis revealed that six proteins, namely PLD3, Synpo, Chrm1, Camk2a, Dcx, and Ezr, were significantly downregulated in the model group but were significantly upregulated after TSS treatment. PLD3 encodes a phospholipase that may play a role in the cleavage and processing of APP, a critical step in the formation of amyloid plaques, which are a hallmark of AD. PLD3 protein may promote the clearance of Aβ through lysosomal degradation pathways. Synpo is reported to be associated with neuroprocesses in mature neurons and can facilitate the degradation of the phosphorylated microtubule-associated protein tau (phospho-MAPT/tau) through autophagy in neuronal processes [43,44]. Further research on these targets showed that abnormally expressed Chrm1 is associated with various neurological disorders, including surgical menopause, Huntington’s disease, and memory function in schizophrenia [45,46,47]. Studies have indicated that increased Chrm1 levels can promote the activation of the cAMP/PKA/CREB pathway. In Aβ-treated SH-SY5Y cells, the upregulation of the cAMP/PKA/CREB signaling pathway after drug treatment inhibited tau hyperphosphorylation and apoptosis. Thus, upregulating Chrm1 can activate the cAMP/PKA/CREB pathway, inhibiting tau hyperphosphorylation and protecting against neurological damage [48]. Chrm1′s involvement in both cAMP and cGMP signaling pathways suggests its potential as a protein marker for AD. Taken together, these findings provide deeper insights into the complex mechanisms underlying AD and may offer potential targets for novel therapeutic approaches.

## 4. Materials and Methods

### 4.1. Preparation of Total Saikosaponins

*Radix bupleuri* was purchased from the Chinese Medicinal Material market in Bozhou, China (Lot No. 20190311). TSS were extracted from *Radix bupleuri* as described in our previous report [18].

### 4.2. Animals and Experimental Design

Nine-month-old male APP/PS1 transgenic mice and wild-type (WT) C57BL/6J mice, each weighing approximately 35 ± 5 g, were obtained from Beijing Huafukang Experimental Animal Technology Co., Ltd., Beijing, China. All animal experiments were conducted with adherence to the guidelines and standard recommendations outlined in China’s “Regulations for the Administration of Experimental Animals” and received approval from the Ethics Committee of Ningxia Medical University (Approval Number: 2020-346). Throughout the duration of the study, the mice were maintained in a sterile environment with controlled conditions, including a 12 h light/dark cycle, consistent temperature (22 ± 1 °C), and humidity (45–55%), with unrestricted access to food and water. The APP/PS1 mice were randomly assigned to two groups (n = 6), a model group and a TSS-treated group. The C57BL/6J WT mice served as the control group. Mice in the TSS-treated group received TSS administered via gavage at a dosage of 80 mg/kg, while the control and model groups were given an equivalent volume of saline as a placebo. The treatment period lasted for 30 days, during which time behavioral assessments, including the Y-maze test, the NOR test, and the Morris water maze test, were conducted from day 22 to day 30. Following the completion of the treatment and the testing period, the mice were euthanized on day 31, and tissue samples were collected for further analysis and evaluation.

### 4.3. Behavioral Tests

After a month of drug administration, the Y-maze experiment was carried out according to the method in [49]. The objective of this study was to assess the short-term memory capacity of AD mice using a Y-shaped maze apparatus. The maze comprised three arms of uniform dimensions, forming an equilateral triangle at their intersection, with a 120-degree angle between any two adjacent arms. Each arm measured 40 cm in length, 10 cm in width, and 15 cm in height. Before the experiment, the maze box was thoroughly wiped with 75% alcohol to remove any residual odor that may affect the mice’s exploratory behavior. During the experimental procedure, the mice were placed in the central area of the Y-maze and allowed to explore freely for a duration of 8 min. The mice could enter the arms labeled A, B, and C in any sequence, with the initial arm choice varying among individuals (e.g., CAB, BCA, ACB, BAC, CBA, and ACB), thereby constituting alternation. The criterion for considering an arm entry as completed was when all four limbs of the mouse had entered the arm. The number and sequence of arm entries for each mouse were meticulously recorded. Based on the collected data, the spontaneous alternation rate was calculated using the following formula: spontaneous alternation = (number of spontaneous alternations/total arm entries − 2) × 100%. This metric served as an indicator of mice’s short-term memory ability, with a higher spontaneous alternation rate suggesting better memory function.

The novel object recognition (NOR) test is a behavioral assay designed to assess an animal’s ability to recognize and distinguish between novel and familiar objects [50]. During the habituation phase of the NOR test, each mouse was placed in a testing box with dimensions of 50 cm × 50 cm × 30 cm and allowed to freely explore for 5 min. Twenty-four hours later, the training session began. During this session, two identical objects were placed in the testing box. Each mouse was then placed in the box and allowed to freely explore for 5 min. The number of touches or explorations of each object was recorded. This phase allowed the mouse to become familiar with the objects. After another 24 h, the test phase began. During this phase, one of the original objects was replaced with a novel object of a similar size but a different shape and color. Each mouse was again placed in the testing box and allowed to freely explore for 5 min. The number of explorations of the novel and familiar objects was recorded. At the end of each experiment, the test box and objects were thoroughly wiped with 75% ethanol to remove any residual odor and prevent it from affecting the exploration of the mice. The data collected during the test phase were statistically analyzed. The discrimination index (DI) was calculated using the formula DI = (TN − TF)/(TN + TF), where TN represents the number of explorations of the novel object, and TF represents the number of explorations of the familiar object. A higher DI indicates better discrimination between the novel and familiar objects, which suggests better cognitive function.

The Morris water maze (MWM) test is one of the most commonly used behavioral experiments for evaluating spatial learning and memory abilities in mice [51]. The MWM test was executed in a circular pool measuring 120 cm in diameter and 40 cm in height, filled with opaque water. A submerged platform, 12 cm in diameter, was placed just 1 cm beneath the water’s surface. The pool was segmented into four quadrants. The experiment lasted for six days, incorporating the place navigation test and the spatial probe test. Across five days of training, place navigation tests were performed to assess mice’s spatial learning ability. Each mouse underwent two training sessions daily, spaced an hour apart. On the sixth day, the spatial probe test was performed to evaluate mice’s spatial memory retention. During this test, the mice were allowed to swim freely in the pool for 60 s. The following data points were documented: the escape latency, which is the time it takes for the mouse to find the hidden platform; the number of times the mice crossed the original platform’s location; and the distance and time spent by the mice within the target quadrant. All experimental data were analyzed using the WMT-100S system developed by Techman Software Co., Ltd., Chengdu, China.

### 4.4. Sample Collection

Following the completion of behavioral tests, six animals were anesthetized and subsequently underwent orbital venous blood collection. Anticoagulation was achieved using heparin. After allowing the samples to stand for ten minutes, they were centrifuged at 3000 rpm at a temperature of 4 °C for 15 min. The supernatant was gathered and preserved at −80 °C for subsequent processing. Once the mouse brains were extracted, they were thoroughly rinsed with pre-cooled phosphate-buffered saline (PBS) and then stored in liquid nitrogen.

### 4.5. Sample Preparation

For metabolomic analysis, the frozen samples stored at −80 °C were thawed in an ice-water bath. A 50 μL aliquot was transferred to a 1.5-milliliter Eppendorf (EP) tube, and 200 μL of a protein precipitation reagent, consisting of methanol–acetonitrile in a volume ratio of 2:1 and containing mixed internal standards at a concentration of 4 μg/mL, was added. The mixture was vortexed for 1 min and then subjected to sonication in an ice–water bath for 10 min. The samples were allowed to stand overnight at −40 °C and subsequently centrifuged at 12,000 revolutions per minute (rpm) and 4 °C for 10 min. Using a syringe, 150 μL of the supernatant was drawn and filtered through a 0.22-micrometer organic phase syringe filter into an LC sample vial, which was then stored at −80 °C until liquid chromatography–mass spectrometry (LC-MS) analysis.

To prepare tissue samples for metabolomic analysis, 30 milligrams (mg) of the sample were weighed into a 1.5 mL EP tube, and two small steel beads were added. Four hundred microliters (400 μL) of a methanol–water solution in a volume ratio of 4:1, containing mixed internal standards at 4 μg/mL, were then pipetted into the tube. The tube was pre-cooled in a −40 °C refrigerator for 2 min before being homogenized in a grinder at 60 Hz for 2 min. The homogenized mixture was sonicated in an ice–water bath for 10 min, left overnight at −40 °C, and centrifuged at 12,000 rpm and 4 °C for 10 min. Using a syringe, 150 μL of the supernatant was aspirated and filtered through a 0.22-micrometer organic phase syringe filter into an LC sample vial, which was then stored at −80 °C until LC-MS analysis. Quality control (QC) samples were prepared by mixing equal volumes of extracts from all samples.

For proteomic analysis, frozen tissue samples were retrieved and thoroughly ground using liquid nitrogen. An appropriate amount of the ground sample was transferred to a 1.5 mL centrifuge tube. Sample lysis buffer, phosphatase inhibitors, and protease inhibitor PMSF were added to achieve a final PMSF concentration of 1 mM. The sample was then subjected to grinding in a chilled grinder at −35 °C and 60 Hz for 120 s, with this grinding process being repeated once. After grinding, the solution was centrifuged at 12,000 rpm and 4 °C for 10 min, and the supernatant, representing the total protein solution of the sample, was collected. The protein concentration was measured using the bicinchoninic acid (BCA) method, and the protein solution was stored at −80 °C for future use.

### 4.6. Non-Targeted Metabolomic Experiment

The analytical instrumentation used in this experiment was an LC-MS system, specifically an ACQUITY UPLC I-Class plus ultra-performance liquid chromatograph coupled with a QE plus high-resolution mass spectrometer equipped with a heated electrospray ionization (ESI) source from Thermo Fisher Scientific (Waltham, MA, USA). This system was used to analyze metabolic profiles in both positive and negative ESI modes.

For separation, an ACQUITY UPLC HSS T3 column with dimensions of 1.8 μm, 2.1 × 100 mm was employed. The gradient elution system used water and acetonitrile, both containing 0.1% formic acid, and followed a specific gradient profile: starting at 5% acetonitrile at 0.01 min; holding at 5% until 2 min; increasing to 30% acetonitrile at 4 min, 50% at 8 min, and 80% at 10 min; reaching 100% acetonitrile at 14 min; and holding at 100% until 15 min before returning to 5% acetonitrile at 15.1 min and holding at 5% until 16 min. The flow rate was set to 0.35 mL/min at 5% acetonitrile, and the column temperature was maintained at 45 °C. All samples were kept at 4 °C during analysis, with an injection volume of 50 μL. The mass spectrometer was operated with a mass range from 100 *m*/*z* to 1200 *m*/*z*, an MS1 scan resolution of 70,000, and an MS2 scan resolution of 17,500. Collision energies were set at 10, 20, and 40 eV. Additional operating parameters included a spray voltage of 3800 V for the positive mode and 3200 V for the negative mode, a sheath gas flow rate of 35 arbitrary units, an auxiliary gas flow rate of 8 arbitrary units, a capillary temperature of 320 °C, an aux gas heater temperature of 350 °C, and an s-lens RF level of 50.

### 4.7. Proteomic Experiments

#### 4.7.1. Protein Digestion, Peptide Labeling, and High-Performance Liquid Chromatography Separation

In this experimental procedure, protein samples from different groups were first normalized to the same concentration and volume using a lysis buffer. DTT was added to achieve a final concentration of 5 mM, and the solution was incubated at 55 °C for 30 min, followed by cooling on ice. Iodoacetamide was then added to a final concentration of 10 mM, and the mixture was incubated in the dark at room temperature for 15 min. Acetone was added to precipitate the proteins, which were collected by centrifugation. The precipitate was redissolved in TEAB, and trypsin–TPCK was added for overnight digestion at 37 °C. The digested samples were lyophilized and redissolved in TEAB buffer. The TMT (iTRAQ) labeling reagent was added to the samples, which were mixed thoroughly and incubated at room temperature for 1 h. The reaction was stopped with 5% hydroxylamine, and the samples were lyophilized and stored at −80 °C. For component separation, liquid chromatography was performed using an Agilent 1100 HPLC equipped with an Agilent Zorbax Extend-C18 column. A gradient elution system was used with mobile phase A (ACN-H_2_O adjusted to pH 10 with ammonia) and mobile phase B (ACN-H_2_O adjusted to pH 10 with ammonia). The gradient elution conditions were specified, and fractions were collected from 8 to 54 min, with repeated sampling in the sequence of 1→15. The fractions were evaporated and stored frozen until ready for mass spectrometry analysis. A total of 15 fractions were collected during the pre-separation step.

#### 4.7.2. Liquid Chromatography–Tandem Mass Spectrometry (LC-MS/MS) Analysis

Each fraction collected from the previous pre-separation step was loaded onto an EASY-nLC 1200 liquid chromatography system for further separation. The system was set to a flow rate of 300 nL/min. Mobile phase A was composed of ACN-H2O-FA in a 99.9:0.1 volume ratio, while mobile phase B was composed of ACN-H2O-FA in an 80:19.9:0.1 volume ratio. The gradient elution conditions specified a gradual increase in the percentage of mobile phase B over time, namely from 8% to 11% B from 0 to 4 min, from 11% to 45% B from 4 to 36 min, from 45% to 100% B from 36 to 39 min, and maintained at 100% B from 39 to 45 min. After separation by ultra-high-performance liquid chromatography, the peptide fractions were injected into a Q Exactive HF mass spectrometer for analysis. The mass spectrometry conditions were configured as follows: the MS1 mass resolution was set to 45,000, the automatic gain control target was set to 3e6, and the maximum injection time was set to 30 ms. The mass spectrometry scan was set to perform a full scan within a mass-to-charge ratio range of 350–1500, and an MS/MS scan was performed on the top 20 most intense peaks. All MS/MS spectra acquisitions were performed using data-dependent high-energy collision dissociation in the positive ion mode, with collision energy set to 32. The MS/MS resolution was set to 30,000, the AGC target was set to 2e5, and the maximum ion injection time was set to 40 ms. The dynamic exclusion time was set to 30 s to avoid repeated sequencing of the same peptide. This process allowed for the identification and quantification of the proteins present in each fraction. The metabolomic and proteomic mass spectrometry data have been deposited in the OMIX, China National Center for Bioinformation/Beijing Institute of Genomics, Chinese Academy of Sciences (https://ngdc.cncb.ac.cn/omix/release/OMIX008611 (accessed on 8 January 2020)).

### 4.8. Data Processing

#### 4.8.1. Preprocessing and Statistical Analysis of Non-Targeted Metabolomic Data

The raw LC-MS data were processed using Progenesis QI V2.3 software, which performed various preprocessing steps including baseline filtering, peak detection, integration, retention time correction, peak alignment, and normalization. The software was configured with specific parameters for precursor and product tolerances, depending on the database used for compound identification. The Human Metabolome Database (HMDB), Lipidmaps (V2.3), Metlin, and a self-constructed database were utilized for compound identification based on the accurate mass-to-charge ratio (*m*/*z*), secondary fragments, and isotopic distributions. After preprocessing, peaks with more than 50% missing values were removed, zero values were replaced with half of the minimum value, and compounds with a low database matching score (below 36 out of 80) were excluded. Positive and negative ion data were combined into a single data matrix.

The data matrix was then imported into an R package for principal component analysis (PCA) to assess the overall distribution of samples and the stability of the analytical process. Orthogonal partial least square–discriminant analysis (OPLS-DA) and partial least square–discriminant analysis (PLS-DA) were employed to differentiate metabolite differences between the groups. To ensure the quality of the models, 7-fold cross-validation and 200 response permutation tests (RPTs) were used. Variable importance in projection (VIP) values from the OPLS-DA model were used to rank the overall contribution of each variable to group discrimination. Additionally, a two-tailed Student’s *t*-test was conducted to verify the statistical significance of differences in metabolites between the groups. Differential metabolites were selected based on a VIP value greater than 1.0 and a *p*-value less than 0.05. These metabolites were considered significant and were further analyzed to understand the biological differences between the groups. The false discovery rate (FDR) was used to control the proportion of false positives in differential analysis, represented by the q-value. All data were calculated using the Benjamini–Hochberg method.

#### 4.8.2. Protein Identification, Quantification, and Functional Analysis

The raw mass spectrometry data were imported into Proteome Discoverer software (Version 2.4, Thermo Fisher Scientific, Waltham, MA, USA) for spectral analysis. The search parameters were set with a parent ion tolerance of 10 ppm; a fragment ion tolerance of 0.02 Da; fixed modifications including TMT (N-term, K) and carbamidomethyl (C); and variable modifications, including oxidation (M) and acetyl (N-term), allowing for a maximum of 2 missed cleavage sites. Differentially expressed proteins were identified by applying a significance threshold of *p* < 0.05 and a fold change (FC) threshold of either greater than 1.2 or less than 1/1.2. Proteins with *p* < 0.05 and FC > 1.2 were considered significantly upregulated, while those with *p* < 0.05 and FC < 1/1.2 were considered significantly downregulated. The false discovery rate (FDR) was used to control the proportion of false positives in differential analysis, represented by the q-value. All data were calculated using the Benjamini–Hochberg method.

To gain insights into the biological functions and classifications of the differentially expressed proteins, Gene Ontology (GO) analysis was conducted using the GO database. This analysis provided information on the biological processes (BPs), cellular components (CCs), and molecular functions (MFs) associated with the proteins. Additionally, the Kyoto Encyclopedia of Genes and Genomes (KEGG) database was used to investigate the major pathways that these differential proteins were involved in. This analysis helped to identify the biological pathways that were significantly affected by the differentially expressed proteins. Protein–protein interaction (PPI) analysis was also conducted based on the STRING database to understand the interactions between the differentially expressed proteins. A PPI network was constructed to visualize these interactions and to identify potential hubs or key players within the network. Collectively, these analyses provided a comprehensive understanding of the differentially expressed proteins, their biological functions, classifications, pathways, and interactions, which can be used to further investigate the underlying mechanisms and potential therapeutic targets associated with the observed changes in protein expression.

#### 4.8.3. Statistical Methods for Behavioral Experiments

The statistical analysis was conducted utilizing GraphPad Prism, version 8, provided by GraphPad Software Inc., San Diego, CA, USA. The data are presented as the mean ± SD. For comparing multiple groups, a one-way ANOVA was employed, followed by Tukey’s post hoc test. Statistical significance was determined as *p*-values less than 0.05.

## 5. Conclusions

**Figure 9:**
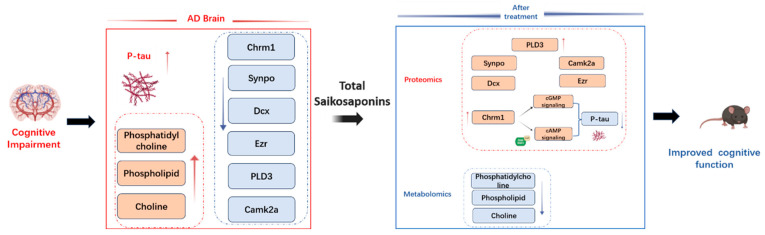
The impacts of TSS on key metabolites and proteins in AD mice. The red arrows indicate upregulation and blue arrows indicate downregulation. The brains of AD mice exhibited the following pathological phenomena: increased tau phosphorylation; elevated levels of phosphatidylcholine, phospholipids, and choline; and downregulation of proteins such as Chrm1, Synpo, Dcx, Ezr, PLD3, and Camk2a. Ultimately, these pathological changes led to cognitive impairment in AD mice. After TSS treatment, metabolomic analysis revealed a decrease in phosphatidylcholine, phospholipids, and choline, while proteomic analysis showed the upregulation of proteins including Chrm1, Synpo, Dcx, Ezr, PLD3, and Camk2a. Additionally, the upregulated Chrm1 can activate the cAMP and cGMP signaling pathways, thereby reducing *p*-tau levels.

As shown in Figure 9, we studied the therapeutic effect and related molecular mechanism of TSS in APP/PS1 mice, and behavioral experiments showed that TSS can effectively improve the learning and memory function of mice. Metabolomic and proteomic analyses suggest that TSS ameliorate cognitive decline by regulating neuroinflammatory pathways, cAMP and cGMP signaling, and synaptic plasticity pathways. Our study provides a robust foundation for further exploration of the specific mechanisms of TSS in AD treatment at both the metabolite and protein regulatory levels. Nevertheless, our current understanding of the interplay between metabolites and associated proteins remains speculative and based on predictions. It is imperative to conduct additional experimental validations to gain a thorough comprehension of the mechanisms driving the therapeutic effects of TSS in AD. These validations will be crucial for advancing our knowledge and potentially leading to new treatments for AD.

## Figures and Tables

**Table 1:** Potential plasma biomarkers associated with short-term administration of TSS in mice.

No.	Metabolites	Model vs. WT	TSS vs. Model
1	PC(16:0/2:0)	down	up
2	PC(18:3(9Z,12Z,15Z)/0:0)	up	down
3	8-Tridecynoic Acid	down	up
4	S-(9-Deoxy-Delta9, 12-Pgd2)-Glutathione	up	down
5	Oob-Pe	up	down
6	1-Nonadecanoyl-Glycero-3-Phosphoethanolamine	down	up
7	Photinus Luciferin	down	up
8	PC(20:5(5Z,8Z,11Z,14Z,17Z)/0:0)	up	down
9	PC(16:0/18:1(12Z)-O(9S,10R))	down	up
10	3,6-Dioxo-Decanoic Acid	down	up
11	PC(18:2(2E,4E)/16:0)	down	up
12	Dodecaprenyl Diphosphate	up	down
13	PC(P-16:0/18:1(12Z)-O(9S,10R))	up	down
14	GM4(d18:1/18:0)	up	down
15	PC(17:1(9Z)/15:0)	down	up
16	PI(18:0/20:4(5Z,8Z,11Z,14Z))	up	down
17	PC(16:1(9Z)/0:0)	up	down
18	2-Tetradecylcyclobutanone	down	up
19	2e,13z-Octadecadienal	down	up
20	Bargustanine	up	down
21	Pentadecanoylcarnitine	up	down
22	PC(19:0/18:3(9Z,12Z,15Z))	down	up
23	Isoquinoline	up	down
24	9,12,15-Octadecatrienal	down	up
25	5′-Carboxy-Gamma-Chromanol	down	up
26	PI(20:1(11Z)/22:6 (5Z,8E,10Z,13Z,15E,19Z)-2OH(7S,17S))	up	down
27	Methylenediphosphonic Acid	down	up
28	GM4(d18:1/20:0)	up	down
29	Octyl Decyl Phthalate	down	up
30	N-Oleoyl L-Serine	up	down
31	Butyramide	up	down

**Table 2:** Potential brain tissue biomarkers associated with short-term administration of TSS in mice.

No.	Metabolites	Model vs. WT	TSS vs. Model
1	PE(PGJ2/22:2(13Z,16Z))	up	down
2	PS(O-20:0/17:1(9Z))	up	down
3	PC(22:6(4Z,7Z,10Z,13Z,16Z,19Z)/20:4(5Z,8Z,11Z,14Z))	up	down
4	DGCC(22:6/20:5)	up	down
5	PE(21:0/17:2(9Z,12Z))	up	down
6	PC(18:2(2E,4E)/18:2(2E,4E))	up	down
7	PC(20:4(5Z,8Z,11Z,14Z)-OH(20)/P-18:0)	up	down
8	Seerziqquaztol-Wojbjxkfsa-N	up	down
9	5s-Hete Di-Endoperoxide	down	up
10	PC(16:0/18:1(11Z))	up	down
11	PE(19:0/18:1(9Z))	up	down
12	PC(17:0/PGE1)	up	down
13	PE(22:5(4Z,7Z,10Z,13Z,16Z)/20:5(7Z,9Z,11E,13E,17Z)3OH(5,6,15))	up	down
14	PS(O-18:0/19:1(9Z))	up	down
15	PC(P-18:1(11Z)/22:5(4Z,7Z,10Z,13Z,19Z)-O(16,17))	up	down
16	PC(20:4(5Z,8Z,11Z,14Z)/16:1(9Z))	down	down
17	PC(14:0/20:1(11Z))	down	up
18	PC(18:1(11Z)/16:0)	up	down
19	PG(20:1(11Z)/20:3(5Z,8Z,11Z)-O(14R,15S))	down	up

**Table 3:** Analysis of related DEPs.

Accession	Gene Name	Regulated Type	log2FC	*p*-Value
P11798	Camk2a	up	0.415196667	0.00623
Q9Z2Y3	Homer1	up	0.380023333	0.027427
Q3U214	Mast3	up	0.28294	0.017301
Q8CC35	Synpo	up	0.58669	0.005549
Q3UQ44	Iqgap2	up	0.301723333	0.015091
P70207	Plxna2	up	0.36823	0.034991
Q9QVP9	Ptk2b	up	0.34146	0.031864
Q9JJV5	Cacng3	up	0.278546667	0.018327
Q8C3Q5	Shisa7	up	0.333286667	0.026621
O35405	Pld3	up	0.366233333	0.021005
Q61909	Runx1t1	down	−0.547753333	0.005818
F6SEU4	Syngap1	up	0.3196	0.025881
A2AF47	Dock11	down	−0.322023333	0.000447
Q64314	Cd34	up	0.51789	0.041513
P49135	Ercc3	down	−0.280473333	0.047553
Q9QZX7	Srr	up	0.34103	0.030063
Q9ERB5	Slco1c1	up	0.26938	0.002302
Q8BIZ1	Anks1b	up	0.372903333	0.033418
P60853	Lzts1	up	0.317206667	0.024648
Q64336	Tbr1	up	0.273523333	0.046988
P14231	Atp1b2	down	−0.487503333	0.004089
Q91XU3	Pip4k2c	up	0.301373333	0.010448
O88343	Slc4a4	down	−0.297656667	0.01442
Q5DTZ6	Rnf150	up	0.289186667	0.007382
Q8BLQ9	Cadm2	up	0.317206667	0.024648
Q9D1T0	Lingo1	up	0.26856	0.002194
Q8BG58	P4htm	down	−0.31637	0.036054
Q76HP3	Tmem132d	up	0.366693333	0.046749
P60761	Nrgn	up	0.46404	0.041644
O70433	Fhl2	up	0.35065	0.001092
P97441	Slc30a3	up	0.364163333	0.044378
Q60987	Foxg1	up	0.337033333	0.031355
Q5NBX1	Cobl	up	0.28674	0.010194
Q6ZPQ6	Pitpnm2	up	0.289453333	0.01947
O88809	Dcx	up	0.303026667	0.013699
Q60625	Icam5	up	0.283706667	0.044947
Q64524	H2bc21	down	−0.51608	0.005131
P97792	Cxadr	up	0.30167	0.03135
Q7TPR4	Actn1	up	0.416243333	0.00801
Q9R257	Hebp1	up	0.301436667	0.035898
P35282	Rab21	down	−0.295966667	0.024016
G5E829	Atp2b1	up	0.278036667	0.04069
P26040	Ezr	up	0.35864	0.048959
P12657	Chrm1	up	0.415196667	0.00623
O35526	Stx1a	up	0.454896667	0.035476
Q9CW07	Ppp1r3g	up	0.448413333	0.000286

## Data Availability

All data supporting the findings of this study are available within the paper and its Supplementary Materials. The metabolomic and proteomic mass spectrometry data reported in this paper have been deposited in the OMIX, China National Center for Bioinformation/Beijing Institute of Genomics, Chinese Academy of Sciences (https://ngdc.cncb.ac.cn/omix/release/OMIX008611 (accessed on 12 January 2025)).

## Supplementary Materials

The following supporting information can be downloaded at: https://www.mdpi.com/article/10.3390/ph18010100/s1, File S1: The ARRIVE Essential 10: Compliance Questionnaire. References [52,53,54,55,56,57] are cited in the Supplementary Materials.

## References

[1] Breijyeh Z., Karaman R. (2020). Comprehensive Review on Alzheimer’s Disease: Causes and Treatment. Molecules.

[2] Long J.M., Holtzman D.M. (2019). Alzheimer Disease: An Update on Pathobiology and Treatment Strategies. Cell.

[3] Busche M.A., Hyman B.T. (2020). Synergy between amyloid-β and tau in Alzheimer’s disease. Nat. Neurosci..

[4] Tripathi T., Kalita P. (2019). Synergistic Effect of Amyloid-β and Tau Disrupts Neural Circuits. ACS Chem. Neurosci..

[5] Chan K.Y., Adeloye D., Asante K.P., Calia C., Campbell H., Danso S.O., Juvekar S., Luz S., Mohan D., Muniz-Terrera G. (2020). Tackling dementia globally: The Global Dementia Prevention Program (GloDePP) collaboration. J. Glob. Health.

[6] Scheltens P., De Strooper B., Kivipelto M., Holstege H., Chételat G., Teunissen C., Cummings J., van der Flier W. (2021). Alzheimer’s disease. Lancet.

[7] Niemantsverdriet E., Valckx S., Bjerke M., Engelborghs S. (2017). Alzheimer’s disease CSF biomarkers: Clinical indications and rational use. Acta Neurol. Belg..

[8] Amft M., Ortner M., Eichenlaub U., Goldhardt O., Diehl-Schmid J., Hedderich D.M., Yakushev I., Grimmer T. (2022). The cerebrospinal fluid biomarker ratio Aβ42/40 identifies amyloid positron emission tomography positivity better than Aβ42 alone in a heterogeneous memory clinic cohort. Alzheimers Res. Ther..

[9] Morris E., Chalkidou A., Hammers A., Peacock J., Summers J., Keevil S. (2016). Diagnostic accuracy of ^18^F amyloid PET tracers for the diagnosis of Alzheimer’s disease: A systematic review and meta-analysis. Eur. J. Nucl. Med. Mol. Imaging.

[10] Rufino-Ramos D., Albuquerque P.R., Carmona V., Perfeito R., Nobre R.J., de Almeida L.P. (2017). Extracellular vesicles: Novel promising delivery systems for therapy of brain diseases. J. Control. Release.

[11] González-Domínguez R., García A., García-Barrera T., Barbas C., Gómez-Ariza J.L. (2014). Metabolomic profiling of serum in the progression of Alzheimer’s disease by capillary electrophoresis-mass spectrometry. Electrophoresis.

[12] Padala S.P., Newhouse P.A. (2022). Blood-based biomarkers in Alzheimer’s disease: A mini-review. Metab. Brain Dis..

[13] Tong Y., Zhao G., Shuang R., Wang H., Zeng N. (2024). Saikosaponin a activates tet1/dll3/notch1 signalling and promotes hippocampal neurogenesis to improve depression-like behavior in mice. J. Ethnopharmacol..

[14] Zhao S., Zhang L., Lian G., Wang X., Zhang H., Yao X., Yang J., Wu C. (2011). Sildenafil attenuates LPS-induced pro-inflammatory responses through down-regulation of intracellular ROS-related MAPK/NF-κB signaling pathways in N9 microglia. Int. Immunopharmacol..

[15] Zeng Q., Li L.F., Siu W.S., Jin Y., Cao M.Q., Li W.F., Chen J., Cong W.H., Ma M., Chen K.J. (2019). A combined molecular biology and network pharmacology approach to investigate the multi-target mechanisms of Chaihu Shugan San on Alzheimer’s disease. Biomed. Pharmacother..

[16] Li Z., Zeng Q., Hu S., Liu Z., Wang S., Jin Y., Li L., Ou H., Wu Z. (2023). Chaihu Shugan San ameliorated cognitive deficits through regulating gut microbiota in senescence-accelerated mouse prone 8. Front. Pharmacol..

[17] Wang Y.Y., Guo Q., Cheng Z.B., Zeng K.W., Liang H., Tu P.F., Chen S.Z., Zhang Q.Y. (2017). New saikosaponins from the roots of *Bupleurum chinense*. Phytochem. Lett..

[18] Li J., Zou B., Cheng X.Y., Yang X.H., Li J., Zhao C.H., Ma R.X., Tian J.X., Yao Y. (2022). Therapeutic effects of total saikosaponins from *Radix bupleuri* against Alzheimer’s disease. Front. Pharmacol..

[19] Kim B.M. (2018). The Role of Saikosaponins in Therapeutic Strategies for Age-Related Diseases. Oxidative Med. Cell. Longev..

[20] Lee T.H., Park S., You M.H., Lim J.H., Min S.H., Kim B.M. (2016). A potential therapeutic effect of saikosaponin C as a novel dual-target anti-Alzheimer agent. J. Neurochem..

[21] Xu B.H., Gao Y.P., Zhan S.H., Xiong F., Qiu W.Y., Qian X.J., Wang T., Wang N.L., Zhang D., Yang Q. (2016). Quantitative protein profiling of hippocampus during human aging. Neurobiol. Aging.

[22] Naveed M., Mubeen S., Khan A., Ibrahim S., Meer B. (2019). Plasma Biomarkers: Potent Screeners of Alzheimer’s Disease. Am. J. Alzheimers Dis. Other Demen..

[23] Mizuno S., Ogishima S., Kitatani K., Kikuchi M., Tanaka H., Yaegashi N., Nakaya J. (2016). Network Analysis of a Comprehensive Knowledge Repository Reveals a Dual Role for Ceramide in Alzheimer’s Disease. PLoS ONE.

[24] Coelho D., Schwartz S., Merino M., Hauert B., Topfel B., Tieche C., Rhiner C., Moreno E. (2018). Culling Less Fit Neurons Protects against Amyloid-β-Induced Brain Damage and Cognitive and Motor Decline. Cell Rep..

[25] Soto-Faguás C.M., Sanchez-Molina P., Saura C.A. (2021). Loss of presenilin function enhances tau phosphorylation and aggregation in mice. Acta Neuropathol. Commun..

[26] Dodiya H.B., Kuntz T., Shaik S.M., Baufeld C., Leibowitz J., Zhang X.L., Gottel N., Zhang X.Q., Butovsky O., Gilbert J.A. (2019). Sex-specific effects of microbiome perturbations on cerebral Aβ amyloidosis and microglia phenotypes. J. Exp. Med..

[27] Dai Z., Hu T., Su S.J., Liu J.M., Ma Y.Z., Zhuo Y., Fang S.H., Wang Q., Mo Z.Z., Pan H.F. (2022). Comparative Metabolomics Analysis Reveals Key Metabolic Mechanisms and Protein Biomarkers in Alzheimer’s Disease. Front. Pharmacol..

[28] Ma X.Y., Huang M., Zheng M.N., Dai C.X., Song Q.X., Zhang Q., Li Q., Gu X., Chen H., Jiang G. (2020). ADSCs-derived extracellular vesicles alleviate neuronal damage, promote neurogenesis and rescue memory loss in mice with Alzheimer’s disease. J. Control. Release.

[29] Du J., Song D., Li Y., Liu J., Huang X., Li B. (2022). Saikosaponin-D Mitigates Oxidation in SH-SY5Y Cells Stimulated by Glutamate Through Activation of Nrf2 Pathway: Involvement of PI3K. Neurotox Res..

[30] Lin H., Zhang C., Gao Y., Zhou Y., Ma B., Jiang J., Long X., Yimamu N., Zhong K., Li Y. (2024). HLH-30/TFEB modulates autophagy to improve proteostasis in Aβ transgenic Caenorhabditis elegans. Front. Pharmacol..

[31] Chen Y., Huang X., Zhang Y.-W., Rockenstein E., Bu G., Golde T.E., Masliah E., Xu H. (2012). Alzheimer’s β-secretase (BACE1) regulates the cAMP/PKA/CREB pathway independently of β-amyloid. J. Neurosci..

[32] Chatterjee P., Lim W.L.F., Shui G., Gupta V.B., James I., Fagan A.M., Xiong C., Sohrabi H.R., Taddei K., Brown B.M. (2016). Plasma Phospholipid and Sphingolipid Alterations in Presenilin1 Mutation Carriers: A Pilot Study. J. Alzheimers Dis..

[33] Lonze B.E., Ginty D.D. (2002). Function and regulation of CREB family transcription factors in the nervous system. Neuron.

[34] Wang X.M., Tang X.L., Li M.T., Marshall J., Mao Z.X. (2005). Regulation of neuroprotective activity of myocyte-enhancer factor 2 by cAMP-protein kinase A signaling pathway in neuronal survival. J. Biol. Chem..

[35] Ricciarelli R., Fedele E. (2018). cAMP, cGMP and Amyloid β: Three Ideal Partners for Memory Formation. Trends Neurosci..

[36] Tong L., Thornton P., Balazs R., Cotman C. (2001). Beta-amyloid-(1-42) impairs activity-dependent cAMP-response element-binding protein signaling in neurons at concentrations in which cell survival Is not compromised. J. Biol. Chem..

[37] Li H., Yang S., Wu J., Ji L., Zhu L., Cao L., Huang J., Jiang Q., Wei J., Liu M. (2017). cAMP/PKA signaling pathway contributes to neuronal apoptosis via regulating IDE expression in a mixed model of type 2 diabetes and Alzheimer’s disease. J. Cell Biochem..

[38] Zhang C., Cheng Y.F., Wang H.T., Wang C., Wilson S.P., Xu J.P., Zhang H.T. (2014). RNA Interference-Mediated Knockdown of Long-Form Phosphodiesterase-4D (PDE4D) Enzyme Reverses Amyloid-β_42_-Induced Memory Deficits in Mice. J. Alzheimer’s Dis..

[39] Shi J., Li Y.Y., Zhang Y., Chen J., Gao J.Q., Zhang T.Y., Shang X.G., Zhang X.N. (2021). Baicalein Ameliorates Aβ-Induced Memory Deficits and Neuronal Atrophy via Inhibition of PDE2 and PDE4. Front. Pharmacol..

[40] Sierksma A., Rutten K., Sydlik S., Rostamian S., Steinbusch H., van den Hove D., Prickaerts J. (2013). Chronic phosphodiesterase type 2 inhibition improves memory in the APPswe/PS1dE9 mouse model of Alzheimer’s disease. Neuropharmacology.

[41] Barger S.W., Fiscus R.R., Ruth P., Hofmann F., Mattson M.P. (1995). Role of cyclic-GMP in the regulation of neuronal calcium and survival by secreted forms of beta-amyloid precursor. J. Neurochem..

[42] Zhang Y., Qian L.L., Liu Y.Y., Liu Y., Yu W.P., Zhao Y.F. (2021). CircRNA-ceRNA Network Revealing the Potential Regulatory Roles of CircRNA in Alzheimer’s Disease Involved the cGMP-PKG Signal Pathway. Front. Mol. Neurosci..

[43] Ji C., Tang M., Zeidler C., Höhfeld J., Johnson G.V. (2019). BAG3 and SYNPO (synaptopodin) facilitate phospho-MAPT/Tau degradation via autophagy in neuronal processes. Autophagy.

[44] Zhang D.-F., Fan Y., Wang D., Bi R., Zhang C., Fang Y., Yao Y.-G. (2015). PLD3 in Alzheimer’s Disease: A Modest Effect as Revealed by Updated Association and Expression Analyses. Mol. Neurobiol..

[45] Pala S., Atilgan R., Kuloglu T., Yalçın E., Kaya N., Etem E. (2021). The decrease in hippocampal transient receptor potential M2 (TRPM2) channel and muscarinic acetylcholine receptor 1 (CHRM1) is associated with memory loss in a surgical menopause rat model. Arch. Med. Sci..

[46] Scarr E., Craig J.M., Cairns M.J., Seo M.S., Galati J.C., Beveridge N.J., Gibbons A., Juzva S., Weinrich B., Parkinson-Bates M. (2013). Decreased cortical muscarinic M1 receptors in schizophrenia are associated with changes in gene promoter methylation, mRNA and gene targeting microRNA. Transl. Psychiaty.

[47] Lee J., Hwang Y., Shin J., Lee W., Wie J., Kim K., Lee M., Hwang D., Ratan R., Pae A. (2013). Epigenetic regulation of cholinergic receptor M1 (CHRM1) by histone H3K9me3 impairs Ca(2+) signaling in Huntington’s disease. Acta Neuropathol..

[48] Zhu G., Fang Y., Cui X., Jia R., Kang X., Zhao R. (2022). Magnolol upregulates CHRM1 to attenuate Amyloid-β-triggered neuronal injury through regulating the cAMP/PKA/CREB pathway. J. Nat. Med..

[49] Liu W., Zhu Y., Wang Y., Qi S., Wang Y., Ma C., Li S., Jiang B., Cheng X., Wang Z. (2017). Anti-amnesic effect of extract and alkaloid fraction from aerial parts of Peganum harmala on scopolamine-induced memory deficits in mice. J. Ethnopharmacol..

[50] Scott K.A., Ida M., Peterson V.L., Prenderville J.A., Moloney G.M., Izumo T., Murphy K., Murphy A., Ross R.P., Stanton C. (2017). Revisiting Metchnikoff: Age-related alterations in microbiota-gut-brain axis in the mouse. Brain Behav. Immun..

[51] Yi M., Zhang C., Zhang Z., Yi P., Xu P., Huang J., Peng W. (2020). β Integrated Metabolomic and Lipidomic Analysis Reveals the Neuroprotective Mechanisms of Bushen Tiansui Formula in an A1-42-Induced Rat Model of Alzheimer’s Disease. Oxidative Med. Cell. Longev..

[52] Hair K., Macleod M.R., Sena E.S., on behalf of the IICARus Collaboration (2019). A randomised controlled trial of an Intervention to Improve Compliance with the ARRIVE guidelines (IICARus). Res. Integ. Peer Rev..

[53] Tihanyi D.K., Szijarto A., Fülöp A., Denecke B., Lurje G., Neumann U.P., Czigany Z., Tolba R. (2019). Systematic Review on Characteristics and Reporting Quality of Animal Studies in Liver Regeneration Triggered by Portal Vein Occlusion and Associating Liver Partition and Portal Vein Ligation for Staged Hepatectomy: Adherence to the ARRIVE Guidelines. J. Surg. Res..

[54] Zhao B., Jiang Y., Zhang T., Shang Z., Zhang W., Hu K., Chen F., Mei F., Gao Q., Zhao L. (2020). Quality of interventional animal experiments in Chinese journals: Compliance with ARRIVE guidelines. BMC Vet Res..

[55] Han S. (2017). A checklist is associated with increased quality of reporting preclinical biomedical research: A systematic review. PLoS ONE.

[56] Chatzimanouil M.K.T., Wilkens L., Anders H.-J. (2019). Quantity and Reporting Quality of Kidney Research. J. Am. Soc. Nephrol..

[57] Leung V., Rousseau-Blass F., Beauchamp G., Pang D.S.J. (2018). ARRIVE has not ARRIVEd: Support for the ARRIVE (Animal Research: Reporting of in vivo Experiments) guidelines does not improve the reporting quality of papers in animal welfare, analgesia or anesthesia. PLoS ONE.

